# Advancements in Nickel-Phosphate/Boron Based Electroless Composite Coatings: A Comprehensive Review of Mechanical Properties and Recent Developments

**DOI:** 10.3390/ma16186116

**Published:** 2023-09-07

**Authors:** Vinod Babu Chintada, Thirumala Rao Gurugubelli, Mohan Rao Tamtam, Ravindranadh Koutavarapu

**Affiliations:** 1 Department of Mechanical Engineering, GMR Institute of Technology, Rajam 532127, Andhra Pradesh, India; vinodbabu.chintada@gmail.com; 2 Department of Physics, School of Sciences, SR University, Warangal 506371, Telangana, India; thirumalaphy@gmail.com; 3 Data Science Lab, Department of Information and Communication Engineering, College of Mechanical and IT Engineering, Yeungnam University, Gyeongsan 38541, Republic of Korea; 4 Department of Robotics Engineering, College of Mechanical and IT Engineering, Yeungnam University, Gyeongsan 38541, Republic of Korea

**Keywords:** electroless Ni-P coatings, composite coatings, multipass coatings, mechanical properties, corrosion

## Abstract

Nickel-Phosphate/Boron (Ni-P/B) electroless coatings have been widely used to improve physical and mechanical properties in various industrial applications, including the automotive, aerospace, chemical processing, food, oil and gas, electronic, textile, and printing industries. Electroless nickel coatings are one of the most popular surface-coating methods due to their low cost and short processing time. The purpose of this review is to look at several coating materials and the existing processes for making electroless coatings on different materials. The improvement of Ni-P/B composite coatings by the incorporation of secondary particles into an alloy matrix at the macro, micro, and nano levels is explained in detail. Process parameters like type of surfactant, annealing temperature, size of the reinforcement material, and reducing-agent percentage on mechanical characteristics like hardness, high-temperature oxidation behaviour, friction, coefficient, wear, and corrosion have been broadly researched and illustrated clearly.

## 1. Introduction

**Figure 1:**
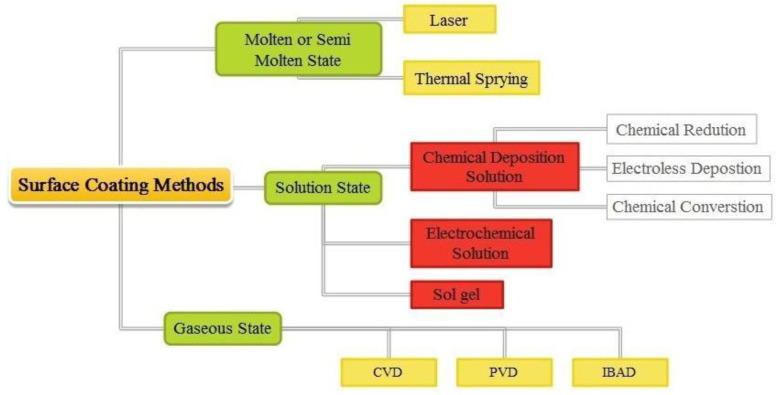
Classification of functional coating.

Surface coating refers to the technique of functionally and aesthetically coating all or part of a material’s surface. To acquire the required mechanical characteristics, a surface is coated with additives, pigments, film-forming substances, and solvents. Functional coatings are utilized in mechanical engineering to provide desired results. These coatings have physical, thermal, optical, electrical, magnetic, and mechanical properties. The numerous kinds of functional coatings are depicted in Figure 1. Gaseous type surface-coating methods are commonly used to coat a thin film where the optical and mechanical properties are desired. The exterior surfaces of the machine element are coated using vacuum deposition techniques in either hard or soft form. Chemical and physical vapour deposition (CVD and PVD) techniques fall under the gaseous state coatings category. To improve the mechanical properties of CVD or PVD, the distance between the gun and the substrate must be much closer [1]. Molten or semimolten state coating methods are utilized to achieve exceptional resistance to the wear and corrosion of structural components. The coating’s main constraint in a molten or semimolten condition is its limited adherence. Electroplating and electroless plating are the two fundamental coating methods used in the solution-state coating process [2,3]. Using an electric current, metal cations are minimized and coated on the electrode surface in the electrochemical deposition method. Compared to most other coating processes, one of the most expensive is electroplating. Electroless deposition is one of the best options out of all the processes because of its unique qualities, such as uniform thickness; this quality makes this process better when compared to other coating processes.

Brenner and Riddell developed electroless Ni plating in 1946 and it has been widely used in several industries since the early 1980s [4]. Without using an electric current, an aqueous solution of nickel alloy is applied to the substrate. Oxidized Ni^2+^ lowers nickel ions and coats the substrate during the autocatalytic electroless nickel-coating process. In a typical process, sodium hypophosphite (NaPO_2_H_2_) is extensively operated as a reducing agent. In some cases, sodium borohydride (NaBH_4_) and dimethylamine borane might be used instead of NaPO_2_H_2_ [5,6]. The main advantages of electroless coating are the inexpensiveness and thickness uniformity of the deposit over complex geometries. These coatings are ideally suited for aluminium, copper, steel, and polymers to achieve greater wear, hardness, and resistance to corrosion. Due to its unusual qualities, it is used as functional coatings in a variety of industries, including chemical, electronics, oil and gas, aerospace, and automobiles. The phosphorus (P) to boron (B)% in the coating determines the characteristics [7,8]. The phosphorus concentration in the coating defines the physical, mechanical, and tribological properties of the nickel-phosphate (Ni-P) coatings. Based on the phosphorus concentration, electroless Ni-P coatings are classified into three groups. The first one is low phosphorus coatings, containing 1–4% of phosphorus and will be used when hardness is desirable. The second is medium phosphorus coatings. The phosphorus concentration in these types of coatings lies between 4–10%. Based on the application, the weight percentage of P is varied; decorative purpose coatings contain 4–7% of P, industrial purpose coatings contain 6–9% of P, and electronic applications contain 4–10% of P. High phosphorus coatings contain a phosphorus weight percentage greater than 10.5% and offer higher resistance to corrosion. These types of coatings are used in highly corrosive acidic environments, such as oil drilling and coal mining [8,9,10,11,12,13].

Ni-B coatings are another essential kind of nickel alloy coatings, where borohydride is considered as a reducing agent. As compared with the Ni-P coatings, nickel–boron (Ni-B) coatings show high hardness, wear resistance, and low corrosion resistance [13,14]. The boron concentration greatly influences the structural and mechanical properties of the coatings. At a low boron concentration, the coatings exhibit a crystalline structure, whereas at a higher concentration, an amorphous structure is observed. The hardness of the coating can be improved by increasing the boron concentration [15,16]. Another important kind of coating is a duplex coating, which is formed by using a dual electroless bath. These offer better corrosion resistance at a high phosphorus concentration and good hardness at low and medium phosphorus concentrations [17]. Electroless composite coatings are formed by the co-deposition of the particulate substances in the metal matrix. Reinforced particles in powder form are added to the electroless bath. Co-deposition of hard ceramic particles such as aluminium oxide, silicon carbides, diamond, and tungsten improve the mechanical properties of the coating. The friction coefficient of the Ni-P coating is reduced by the reinforcement of lubricating particles such as graphite, PTFE, and molybdenum disulfide [18,19,20].

This review focuses on recent achievements in electroless coating research that provide a clear roadmap for future breakthroughs. Ni-P/Ni-B and its composite coating fabrication techniques and modification tactics have been thoroughly covered in this paper. The first part of the review mainly focuses on the recent research work done in Ni-P coatings and their composites. Earlier, researchers tried various efforts to enhance the corrosion, tribological, and mechanical properties of Ni-P coating. The review also focuses on the influence of pH and surfactant type on the properties of the Ni-P composite coatings. The recent advancement in poly alloy coatings is also described in detail. The current review additionally looks at other electroless Ni-B and their composite coatings as well as multi pass and Cu-P coatings. Finally, conclusions and the future advancement in electroless Ni coatings based on literature are described.

## 2. Coating Formation Mechanism

The materials to be coated are immersed in an electroless (EN) solution during the electroless coating process. Nickel chloride (NiCl) and nickel sulphate (NiSO_4_) are utilized as metal ion bases in an electroless nickel solution. NaPO_2_H_2_ and NaBH_4_ are used to generate Ni-P and Ni-B coatings, respectively, depending on the type of coating required [5,6]. To escape the breakdown of the electroless bath and to control the reactions, organic salts are utilized as complexing agents. To speed up the deposition rate and separation of hydrogen from the NaPO_2_H_2_/NaBH_4_, a diluted accelerator is added to the chemical bath [8,9]. An additive and buffer are added to the suspension to stabilize it and maintain a constant pH throughout the process. By adding second-phase soft particles and hard ceramic particles to the chemical bath, the EN composite coating is produced. Finally, the chemical bath is sustained at 85–95 °C for a period of 2–4 h to achieve the desired thin film. However, the duration of time is essentially upheld in order to achieve the appropriate coat thickness while maintaining a consistent deposition rate [21,22,23,24,25,26].

Fabrication of electroless Ni and Ni composite coatings by reinforcing different hard and soft particles to the Ni matrix to exploit the coating performance to meet the demanding requirements of engineering applications is discussed in detail in this paper. The process of creation, parameters that influence particle co-deposition, the result of particle inclusion on the coating structure, friction, hardness, abrasion resistance, resistance to wear and corrosion, and applications are all explored [10,14]. This article goes through single-pass, composite, and multi pass electroless coatings in depth. Electroless Ni coatings are a combination of nickel and phosphorus or bromide. The presence of phosphorus and bromide in the coating mainly depends on the selection of a reducing agent. To produce the Ni-P/B coatings, hypophosphite and borohydride are chosen as reducing agents. The first part of the literature mainly focuses on the recent research work done in Ni-P coatings and their composites. Earlier, researchers tried various efforts to enhance the corrosion, tribological, and mechanical properties of Ni-P coating. Several mechanisms are proposed for chemical reactions in hypophosphite electroless baths. The most widely accepted mechanisms are illustrated below [21,27]. Oxidation of the hypophosphite gives up electrons at the catalytic surface and reduces nickel and hydrogen ions.

$$H_{2}PO_{2}^{-}+H_{2}O\to H_{2}PO_{3}^{-}+{2H}^{+}+{2e}^{-}$$

$${Ni}^{++}+{2e}^{-}\to Ni$$

$${2H}^{+}+{2e}^{-}\to H_{2}$$

$$H_{2}PO_{2}^{-}+{2H}^{+}+e^{-}\to P+{2H}_{2}O$$

The atomic hydrogen mechanism involves the separation of hydrogen atoms from hypophosphite by catalytic dehydrogenation.

$$H_{2}PO_{2}^{-}+H_{2}O\to HPO_{3}^{--}+H^{+}{2H}_{ads}$$

$${{2H}_{ads}+Ni}^{++}\to Ni+{2H}^{+}$$

$$H_{2}PO_{2}+H_{ads}\to H_{2}O+OH^{-}+P$$

## 3. Ni-P–Ni-P Composite Coatings

### 3.1. Ni-P Coatings

The pickling method, annealing process, complexing agent concentration, electroless bath pH value, and operating temperature all affect the Ni-P coating’s properties. The amount of phosphorus (P) in the deposit influences its microhardness and corrosion characteristics. Increased phosphorus in Ni-P coatings reduces the corrosion rate and hardness. The amount of “P” in the coating is mainly determined by the deposition time, reducing agent, complex agent concentration, and electroless bath pH value. By increasing the coating period from 0.5 h to 3.5 h, the hardness of the coating rises to 30%. The microhardness of the film increases as the “P” content falls with the increasing deposition time [21,22,23,24]. The concentration of phosphorus in the coatings can be adjusted by altering the complex agent (lactic acid and acetic acid) ratios in the coating solution. The coating deposition rate increases as the lactic acid proportion increases, with a maximum deposition rate of 16.4 mg/cm^2^h recorded at a donor atom proportion of lactic acid to acetic acid of 4/6. The phosphorus concentration in a Ni-P coating increases with the increasing donor-molecule ratio of lactic acid to acidic acid [25].

Increasing the pH value of the solution up to 7.2 reduced the phosphorus deposition (3.75 wt%) in the coating. At a bath pH of 8.2, the coating has the maximum P content (13.48 wt%). The concentration of phosphorus in the coatings determines their thickness. In an acidic or neutral bath, low and medium phosphorus EN coatings with a thickness of 13.9–19.8 m is produced. A 4.8–5.8 μm thickness range is observed in the coating formed in an acidic bath [26]. The decrease in the phosphorus concentration with the increasing pH value changes the amorphous nature of the coating into a mixture of amorphous and nanocrystalline phases. A decrease in P content increases the microhardness value and a maximum value of 634 HV is achieved at a P concentration of 3.67 wt% [28]. The electroless bath temperature influences the properties of the coating. At 60 °C, the bath operating temperature coating has a cauliflower-like structure. The coating’s nodular size grows as the bath temperature rises, and a more compact structure is observed at 80 °C, resulting in superior properties to the coating [29,30].

On aluminium alloy, a coating generated at 70 °C bath temperature enhances the substrate corrosion potential by 0.29, which confirms the rise in corrosion impedance of the 6061 Al alloy [25]. The rate of corrosion of the Ni-P coating is less than the aluminium 7075 T6 alloy. The rise in the phosphorus concentration significantly enhances the impedance to corrosion of Ni-P coatings. While testing corrosion, the development of phosphide film in high-phosphorus Ni-P coatings inhibit hydration at the electrode surface, resulting in the coating having higher corrosion resistance [31]. Ni-P coatings on magnesium alloy (Mg-Zn-Zr denoted as ZK60) primarily possessed crystalline structures, with a small microcrystalline structure in the coating on magnesium alloy (denoted as ME2O alloy). As a result, the Ni-P coating on the ZK60 was more adhesive and corrosion resistant than that on the ME20 alloy [32]. Li et al. identified that a Ni-P coating produced at a pH level ranging from 3.5 to 6 gave maximum corrosion resistance to A3 steel [33]. The thickness and microhardness of nano silicon carbide and Ni-P coatings were maximum when the electroless solution pH was 6–7 and the temperature was 85°C [34]. When compared to thiourea stabilizer, the phosphorus deposition rate (10.68 wt%) in the Ni-P coatings is greatest in the occurrence of lead acetate stabilizer, resulting in the highest resistance to corrosion [35]. The inclusion of potassium iodate stabilizer improved the bath’s life cycle and stability. Additionally, the smoothness and density of the Ni-B coating were greatly enhanced [36].

The Ni-P coating characteristics and microstructure are affected by the deposition period and type of pickling procedure (1* 125 g/L Cr_2_O_3_ and 110 mL/L HNO_3_ (w = 68%) 2* 180 g/L Cr_2_O_3_ and 1 g/L KF). The adhesion of the coating is high in the pickling process 1*. A higher phosphorus amorphous coating formed with the pickling process 1* offers better resistance to corrosion and minimum hardness than the pickling process 2*. A Ni-P coating formed with the pickling process 2* offers better hardness due to the high plastic deformation resistance of the coating at a low phosphorus concentration. At high phosphorus concentrations, the coating becomes more compact and pore free, which results in better corrosion resistance with pickling 1* [37]. By delaying the pickling period, the corrosion resistance of the EN deposit is increased, and subsequently, it is gradually reduced. As the pickling period increases, the rate of corrosion of the Ni–P coating decreases. The coating with the lowest current density and best corrosion resistance was observed with a pickling period of 120 s and a dense structure and smooth surface [38]. On pipeline steel, the Ni-P coating deposition rate increases up to 4 h of plating time, with a maximum deposition rate of 15 m/h observed. The deposition rate gradually decreases when the deposition time increases up to 8 h. Beyond this time, the deposition rate rapidly decreases to 1 μm/h due to the depletion of the chemical solution. A Ni-P coating significantly improves surface hardness because of its brittle nature. The coating’s brittle character is confirmed by lower plastic deformation during the indentation test [39]. The EN Ni-P coating gives significant corrosion resistance to the low-Cr X20CrMoV12-1 steel, as compared to the advanced Ni alloy 230 [40]. Due to their low cost and ease of use, electroless nickel-plating processes are widely utilized for the metallization of plastic parts and beechwood surfaces [41,42].

#### Effect of Heat-Treatment Temperature on Properties of the Ni-P Coatings

Annealing temperature and time have a marked effect on the crystal structure and properties of the deposit. Significant structural transformations were found in Ni-P coatings at different heat-treatment temperatures ranging from 200 to 700 °C [43,44]. Lower annealing temperatures on the order of 200 °C have a slight influence on the microhardness of the EN coating due to incomplete phosphide formation. The formation of nickel phosphide (Ni_3_P) at temperatures ranging from 350 to 400 °C greatly enhances the coating’s microhardness. The Ni_3_P phase formation at heat treatment above 400 °C retards the hardness of the coating. Consequently, at a 500 °C annealing temperature, the coating has a lower microhardness value. In addition to the annealing temperature, the annealing time also influences the structure of the coating [45,46]. The structure of the coating is not much affected up to 12 h of annealing time and at a 200 °C annealing temperature. An increase in amorphous Ni-P cluster size above 12 h of annealing time improves the coating’s microhardness. When the coating was thermally processed at 400 °C for 1 h, structural changes from amorphous to crystalline Ni and Ni_3_P occurred, resulting in increased microhardness and corrosion resistance. It is described that the friction coefficient of heat-treated (at 320 °C and 400 °C) Ni-P coatings is 17% less than the hard chromium coatings [47]. After annealing, the formation of an oxide layer acts as a natural lubricant at the mating surface, reducing the coefficient of friction. As the annealing temperature rises, so do the friction coefficient, resistance to wear, and corrosion. Significant resistance to wear and corrosion is observed in the coatings at annealing temperatures of 400 °C and 600 °C [48,49]. Kundu et al. identified that the friction coefficient of Ni-P coating increases up to 100 °C test temperatures, but thereafter, it decreases due to the growth of the oxide layer. The wear rate of the coating increases up to 500 °C test temperature. Beyond this value, a decrease in wear rate is observed [50].

### 3.2. Electroless Ni-P Composite Coatings

Different hard and soft particles were reinforced in a Ni matrix to form Ni-P composite coatings. Adding different particles of different sizes (macro, micron, and nano) to the Ni-P lattice enhances the tribological and mechanical properties of the coating [51]. The co-deposition of several particles, such as SiO_2_, WC, Si_3_N_4_, ZrO_2_, ZnO, TiO_2_, SiC, Al_2_O_3_ and diamond, increases the coatings’ hardness, resistance to wear, and corrosion. The addition of molybdenum disulfide, polytetrafluoroethylene (PTFE), and graphite lubricating particles reduces the coating’s friction coefficient.

The rate of reinforcement of particles into the alloy lattice determines the composite coating’s quality and properties. The deposition rate of co-deposited particles depends on the amount of secondary particles in the electroless bath, the agitation technique, and the kind of surfactant utilized in the deposition process. The co-deposited particle concentration in the deposited film is improved by increasing the particle concentration in the plating solution up to an optimum level. Beyond the critical concentration, the particle deposition rate in the deposit decreases due to the agglomeration of second-phase particles in the chemical bath. Therefore, the optimal particle concentration should be selected to prevent agglomeration [52,53]. Abdel et al. discovered that the number of Al_2_O_3_ nanoparticles (NPs) co-deposited in the coating rises to alumina concentrations of 70 g/L in the bath [54]. In the Ni-P composite coatings, the highest number of SiC NPs is uniformly reinforced at 2 g/L SiC concentration [55]. In the coating, the deposition rate of particles depends on the dispersion rate in the electroless solution. The agitation technique utilized in the coating process influences the particle distribution rate in the plating solution. Mechanical stirring, magnetic stirring, air bubbling, and ultrasonication agitation techniques are used for better particle dispersion in the electroless solution. In the ultrasonic agitation method, particles are more homogeneously dispersed in the electroless bath compared to the nitrogen bubbling, mechanical, and magnetic stirring techniques [56,57]. Xiang et al. tried the nitrogen, mechanical, and injection agitation techniques [58]. They found that the dispersion rate of the diamond NPs is at a maximum with the injection agitation method.

The number of secondary particles reinforced into the coating is largely determined by the nature of the surfactant used in the electroless process. The wettability is improved by the surfactant, which also increases the stability of the reinforced particles. The particles’ net positive charge increases with the existence of surfactant, enhancing the particles’ absorption rate on the cathode surface. As a result, the choice of surfactant is critical in preventing nanoparticle agglomeration [59]. The type of surfactant used in the electroless process has a significant effect on the ability of particles to deposit in the composite matrix. The weight percentage of aluminium oxide in the coating is doubled in the presence of a nonionic surfactant compared to the absence of a surfactant. The zeta potential of Al_2_O_3_ NPs increases with the increasing amount of CTAB cationic surfactant in the electroless solution. An increased zeta potential strengthens the electrostatic repulsive force between particles, preventing particle aggregation. As a result, the meditation of Al_2_O_3_ in the coating increases up to 20 g/L. Beyond these values, free CTAB molecules are deposited first on the cathode surface, reducing the deposition rate of Al_2_O_3_ in the coating [4,60]. In the presence of DTAB cationic surfactant, the deposition rate of ZrO_2_ and TiO_2_ NPs is higher than that of anionic and nonionic surfactants. By reducing surface tension and improving wettability, a rise in surfactant concentration accelerates the deposition of alloying particles [61,62]. In the presence of nonionic surfactants, the diffusion rate of PTFE particles is higher. Fluorinated alkyl quaternary ammonium iodides, a surfactant, can be concentrated up to 200 mol/L without an increase in PTFE particle deposition [4,59,60,61,62,63]. The inclusion of an anionic surfactant increases the reinforcement of CNT and TiC NPs in the coating compared to cationic and polymeric surfactants. By increasing the concentration of ionic surfactant, the deposition rate of nano-TiC particles is accelerated. CNT distribution in the Ni-P coating is more uniform at the optimal concentration of the anionic surfactant (SDS) ratio (2 g SDS to 25 mg CNT ratio) due to the higher dispersive capacity of SDS [64,65]. In comparison to anionic surfactant (SDS), the deposition rate of SiC particles at various concentrations (0–200 g/L) is highest in the incidence of cationic surfactant [66].

#### 3.2.1. Properties of the Electroless Ni-P Composite Coatings

##### Microstructure

The weight percentage of phosphorus in the deposit affects the microstructure. Therefore, the properties of the coating change according to the phosphorus concentration. Amorphous, crystalline, and a combination of amorphous and crystalline structures are observed in low-, medium-, and high-phosphorus coatings. In addition to the phosphorus concentration, various factors such as co-deposited particles, annealing temperature, and time influence the structure of the coatings. Co-deposition of Si_3_N_4_, TiO_2_, and CeO_2_ particles has no significant effect on the coating’s microstructure [67,68]. Similar XRD reports of Ni-P-Si_3_N_4_ and Ni-P coatings demonstrate that the co-deposition of Si_3_N_4_ particles has no effect on the coating structure. The incorporation of SiC and B_4_C particles into the Ni lattice alters the coating’s microstructure. The crystalline character of the coating increased after the reinforcement of SiC particles, resulting in an increase in microhardness [69,70]. The annealing process altered the amorphous phase of the coatings into crystalline Ni and Ni_3_P, which improves the coating’s characteristics even further. Higher crystallization is noticed in the coating that has been heat treated at 400 °C [71,72].

##### Porosity

The corrosion resistance of the coating mainly depends on the porosity. Nonporous surface structures provide better anticorrosion properties. A nonporous surface reduces the interaction of the metal with the corrosion solution, increasing the corrosion resistance of the coating [73]. The porosity of the coating is mainly determined by the incorporation rate of the particles in the alloy matrix and the thermal process temperature. The increased co-deposition rate of SiO_2_ and TiO_2_ NPs reduces the holes in the Ni alloy and makes the deposit denser [74,75]. Grain refinement caused by the heat-treatment technique also minimizes the number of micropores in the coating.

##### Microhardness

Inserting hard ceramic particles into the deposited Ni matrix increases the microhardness. The coating hardness is also affected by the particle co-deposition rate, wt% of P, and annealing temperature. The homogeneous distribution of particles all through the Ni lattice leads toan enhancement in the coating’s microhardness. Ni-P coating hardness on mild steel surface is enhanced to 13% by the Al_2_O_3_ NP’s incorporation into the nickel lattice [76]. In the electroless solution, at the optimum concentration of the reducing agent, high deposition of alumina NPs is seen in the coating. The increased reducing-agent concentration raises the phosphorus content in coatings, resulting in a loss in hardness due to the growth in the coating’s amorphous phase. Annealing at 400 °C for a Ni-P-Al_2_O_3_ coating ensures a 135% improvement in microhardness due to the formation of hard crystalline Ni and Ni_3_P phases. An increase in hardness minimizes the coating-specific wear rate [27,77]. The coating microhardness on the copper surface was 27% higher after incorporating Al_2_O_3_ NPs into the Ni-P lattice. The coating microhardness on a copper surface is higher by 27% after the inclusion of Al_2_O_3_ NPs into the Ni-P lattice. To prepare Ni-P composite coatings, SiC particles are used as reinforced materials due to their exceptional chemical stability, abrasion, and shear resistance. The growth of SiC particles in the plating bath increases the concentration of SiC particles in the deposit up to this limit, which improves the microhardness of the coating up to the optimum concentration. Beyond this value, no change in microhardness is observed due to the nickel matrix’s inability ability to support SiC particles at higher concentration [71,78]. Thermal processes improve the microhardness of the Ni-P-SiC coating. A stable crystalline phase development leads to a slight increase in microhardness at an annealing temperature of 200 °C. The maximum hardness value in the coatings is observed at a temperature of 400 °C due to the development of Ni_3_P and Ni intermetallic complexes. Above 400 °C, the reaction between the SiC particles and the matrix results in the development of microscopic cohesive particles that gradually reduce the microhardness [55,79].

Carbon nanotubes (CNT) have received increased attention in recent years due to their inimitable structure and innovative qualities like excellent load-transmit resistance and electrochemical properties. By reinforcing CNTs into the Ni lattice, the microhardness of the deposit increases by 42% [80]. SWNTs are extremely stiff and robust carbon nanotubes that are utilized as reinforcement materials in EN coatings. The maximum deposition of SWNTs recognized in the coating occurs at the optimum concentration of SWNTs (1 wt%) in the electroless bath, resulting in increased hardness for the Ni-P-SWNT coatings. At 400 °C annealing temperature, the coating has a higher microhardness of 1204 HV [81]. CNTs have the highest load-transfer resistance and self-lubricating capabilities than SiC particles. Accordingly, the Ni-P-CNTs had the utmost microhardness [82]. In the Ni-P-Si_3_N_4_ and Ni-P-SiO_2_ coatings, hard crystalline Ni_3_P phase establishment is higher at an annealing temperature of 400 °C than at 200 and 600 °C, resulting in maximum hardness. Nominal phase transmission results in a negligible improvement in coating microhardness at a 200 °C annealing temperature. At 600 °C annealing temperature, coarse grain formation makes the coating brittle and lowers the microhardness [68,83]. Ni-P-Si_3_N_4_ coatings are softer due to the lower adhesive strength of Si_3_N_4_ NPs to the alloy lattice. Formation of the Ni_3_P phase after annealing considerably enriches the hardness of the Ni-P-Si_3_N_4_ coatings [84]. A Ni-P-rGO coating has a hardness value of 761 HV on low-carbon steel [85]. The inclusion of TiN particles into the Ni alloy lattice enhances the Ni-P coating microhardness by 33%. The microhardness of Ni-P-TiN coatings improves by 90% after the thermal process [86]. The weight percentage of ZnO particles in the electroless solution significantly affects the coating properties. The Ni-P-ZnO coating developed at optimal ZnO particle concentration (0.5 g/L) enhances the mild steel-surface microhardness by 60% [87,88].

**Figure 2:**
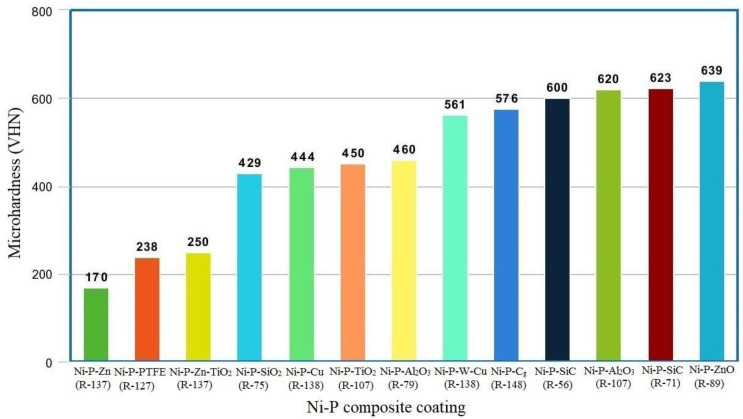
Microhardness of the various Ni-P composite coatings formed at optimum conditions.

In comparison to Ni-P coatings, a Ni-P-TiO_2_ coating produced on 211Z Al alloy by mixing a TiO_2_ solution into the coating bath provides a 13% higher microhardness. The microhardness of the composite coating improves when more boundaries form as a result of the production of fine spherical nodular-shaped grain after the introduction of TiO_2_ particles. Due to a phase shift from disordered face-centered cubic Ni to crystalline Ni_3_P and ordered face-centered cubic Ni to crystalline Ni_3_P, the post-heat-treatment method increases the microhardness by 94% [89]. Maximum microhardness was noticed in the Ni-P-TiO_2_ coating formed on aluminium alloy with an optimal amount of TiO_2_ (5 g/L) particles [90]. A higher degree of crystallization and small Ni_3_P crystallite size at 400 °C annealing temperature retains plastic deformation under loading, resulting in an increase in Ni-P-W coating microhardness [72,91]. Figure 2 depicts the microhardness of several EN coatings created under optimal conditions. It confirms that, at the optimum working conditions of an electroless bath, the Ni-P composite coating generated by reinforcing the hard ZnO, Al_2_O_3_, SiC, W, and Cg nanoparticles has a greater microhardness. The harder coating provides good wear resistance. Since the Ni-P composite coating is harder, it can be employed in industries such as automobiles, chemicals, and textiles that demand a coating with a low wear rate.

##### Wear Resistance

Hardness determines resistance to wear. Consequently, the hard Ni-P composite coatings are resistant to wear. The maximum hardness reported in the coatings at 400 °C annealing temperature demonstrates that the annealing process improves the coating’s wear resistance. The formation of intermetallic products, such as Al_3_Ni, Al_3_Ni_2_, nickel silicide, and crystalline Ni_3_P at an annealing temperature of 400 °C/h, improves hardness, wear, and adhesion of the Ni-P-Al_2_O_3_/SiC coating [79,92]. Compared to the Ni-P coating, the presence of Al_2_O_3_ NPs in the Ni-P-Al_2_O_3_ coating during the wear test limits the promotion of microcracks, resulting in higher wear resistance. Superior wear resistance and hardness were observed in a Ni-P-Al_2_O_3_ film annealed at 400 °C temperature [93]. Reinforcement of graphite, CNT, and nano-SiC particles into Ni-P coatings increases the friction coefficient and reduces weight during the wear test. The highly hardened and wear-resistant CNT and SiC NPs act as a secondary barrier in the alloy lattice, delaying the plastic deformation of the coating and increasing its hardness and wear resistance. Under oil-lubrication conditions, the CNTs in the Ni matrix control the dislocation motion of the alloy matrix, resulting in the lowest cost in Ni-P-CNT coatings [70,94]. Ni-P coatings, TiN, and SiC particle reinforcement minimize the wear loss [95,96]. This is due to the increased hardness of the coating as a result of the addition of TiN and SiC particles. The ideal ratio for reinforcing particles to produce a homogenous Ni-P-SiO_2_-MoS_2_ covering was found to be 7 g/L. Combining the hard Ni_3_P and SiO_2_ phases has boosted the wear resistance following the heating treatment [97].

Mukhopadhyay et al. concluded that the depth of wear in the Ni-P-W is typically influenced by the sliding speed pursued by the sliding interval and applied load for both dry and lubricating conditions [98]. A Ni-P-PTFE coating provides a small friction coefficient and superior wear resistance due to good lubrication and poor mechanical hardness of PTFE particles [99]. The NiSO_4_, NaPO_2_H_2_, and CuSO_4_ content used in the EN bath and annealing temperatures all influence the wear properties of the Ni-P-Cu coating. The coating’s wear depth decreases when the copper sulphate-reducing-agent concentration and heat-treatment temperature increase, but it increases as the nickel supply increases [100]. The addition of AlN particles increased the hardness of the Ni-P coating and improved its wear resistance. After performing annealing on the nanocomposite coating, the hardness value is improved by 12%. When compared to the other samples, the identical sample showed a lower wear rate [101]. The hardness of the Ni-P-MOF coating is superior to that of the Ni-P alloy coating before and after annealing, resulting in better wear resistance [102].

##### Corrosion Resistance

Due to their low porosity and thickness uniformity, the corrosion rate of Ni-P composite coatings is lower than that of Ni-P coating. Coating corrosion resistance is a function of phosphorus content and particle composition. By floating the weight percentage of phosphorus in the thin film, the level of corrosion resistance is improved. Loading NPs decreases the porosity of the coating, resulting in improved corrosion resistance. The incorporation of TiO_2_ NPs and CNTs into Ni-P coatings reduces the corrosion current density when the coatings are coated and heat treated, which ensures improved corrosion resistance of the coating. After annealing, the grain size of the Ni-P-TiO_2_ coating decreases from 28 nm to 17 nm, which increases the corrosion resistance of the coating [57,75]. Up to 11.8 wt% of Ti, a rise in resistance to corrosion was noticed in the Ni-P-Ti coatings before and after annealing, then decreases somewhat when 15.2 wt% Ti [103] is reached. Ti in Ni-P-Ti coatings acts as a corrosion barrier and provides improved corrosion protection for AISI 1018 steel [104]. Lee et al. examined the corrosion rate in two different Ni-P-TiO_2_ and Ni-P-CNT coatings and found the best performance in high phosphorus Ni-P-CNT coatings [105]. Adequate reinforcement of nano-SiO_2_ (2 g/L) and Si_3_N_4_ (5 g/L) particles into the coating lattice eliminates porosity through refinement of the nodular structure of the Ni-P composite coating, further enhancing corrosion resistance [106,107]. Ni-P-CNT coatings developed at a 10 mg/L CNT concentration demonstrated superior resistance to corrosion in the longer corrosion-environment exposure time. The optimum requirement of CNTs changes with the corrosive environment exposure time [108].

The retardation in the corrosion current density by reinforcing Si_3_N_4_ particles instead of Al_2_O_3_, TiO_2_ and CeO_2_ into the Ni-P matrix demonstrates that the corrosion performance of the Ni-P-Si_3_N_4_ coating is superior to that of the Ni-P-TiO_2_/CeO_2_O_3_ coatings. The retardation in the corrosion current density by the reinforcement of Si_3_N_4_ particles instead of Al_2_O_3_, TiO_2_, and CeO_2_ into the Ni-P matrix demonstrates that the Ni-P-Si_3_N_4_ coating corrosion performance is superior to the Ni-P-TiO_2_/Al_2_O_3_/CeO_2_ coatings. The resistance to corrosion of the coatings follows the order: Ni-P-Si_3_N_4_ > Ni-P-CeO_2_ > Ni-P-TiO_2_ > Ni-P-Al_2_O_3_ [109,110]. The Ni-P-coated AZ91D magnesium alloy tribo-chemical behaviour is enhanced by the reinforcement of TiO_2_ into the coating lattice [111]. As deposited conditions, the polarization resistance (549 cm^2^) and charge-transfer resistance (996 cm^2^) of the Ni-P-ZrO_2_-CeO_2_ coating are stronger than those of the Ni-P coating, indicating better resistance to corrosion. The coating performance was greatly improved by annealing at 350 °C temperature [112]. The integration of NiTi particles into the Ni lattice inhibits fracture initiation and transmission during the scratch test, minimizes the surface area for localized corrosion, and improves the Ni-P coating resistance to corrosion [104].

**Figure 3:**
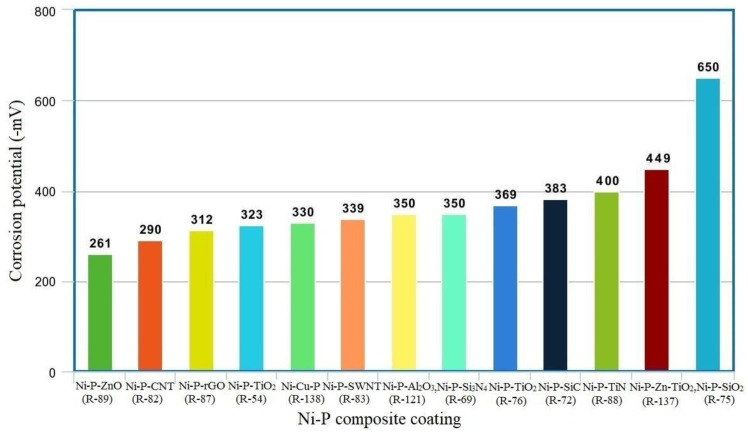
Corrosion resistance of the electroless Ni-P composite coating formed at optimum conditions.

The uniform distribution of SiC and TaCNPs throughout the composite coating avoids the occurrence of pitting during the erosion test; as a result, the coating’s cavitation erosion resistance improves. At the optimum annealing temperature (400 °C), uniformly dispersed nanoparticles in Ni-P coatings create an additional coherent boundary with the Ni lattice, resulting in a pore-free Ni-P coating. Therefore, the resistance to corrosion of the Ni-P composite coating is greatest at 400 °C heat-treatment temperature [113,114]. The Ni-P-SiC coating produced at the concentration of SiC is 1 g/L, providing good resistance to corrosion to the AZ 31 magnesium alloy. The annealing process at 300 °C minimizes the rate of corrosion in the Ni-P-SiC coating even further [96]. SiC NPs’ electrochemical resistivity is greater than that of ZrO_2_ and TiO_2_ NPs. Consequently, the corrosion performance of the Ni-P-SiC coating is higher than that of the other two coatings as deposited and after annealing [115]. Figure 3 shows a comparison of the corrosion potential of various coatings as deposited conditions. Positive shifting of the coating’s corrosion potential is strong proof of the coating’s greater corrosion resistance. This demonstrates Ti, TiO_2_, and ZnO nano particles’ superior corrosion resistance. The corrosion resistance of carburetor and fuel injection systems used in the automobile sector is required during alternative fuel usage. Similarly, corrosion protection is required for hydraulic actuator splines, landing-gear components, engine mounts, engine-oil feed tubes, and other components utilized in the aircraft industry. According to the findings of the preceding investigations, Ni-P composite coatings provide good corrosion resistance.

## 4. Influence of pH and Surfactant Type on the Properties of the Ni-P Composite Coatings

The pH value of the electronic solution and the nature of the surfactant used in the EN coating process have significant effects on the properties of the Ni-P alloy coating.

### 4.1. Influence of Electroless Bath pH

The electroless bath pH value has a considerable influence on an EN coating’s microhardness and corrosion rate. An increase in the electroless solution pH value increases the deposition rate and, on the other side, reduces the phosphorus wt% in the coating. Phosphorus-content reduction enhances the microhardness while decreasing the protection from corrosion [116]. For Ni-P-Al_2_O_3_ coatings, the deposition rate of Al_2_O_3_ particles is maximum at electroless bath pH values ranging from 5–7. The Ni-P-Al_2_O_3_ coating-deposition rate is similarly highest in this range. Therefore, Ni-P composite coatings generated at a pH range of 5–7 provide the highest microhardness values [117,118]. G. Yin-Ning et al. noticed that the resistance to wear and corrosion is maximum to the Ni-P-SiC coating obtained at a 5.2 electroless bath pH value. On mild steel substrates, Ni-P-ZnO/Al_2_O_3_coatings are developed by altering the pH of the chemical solution (9, 11, and 13) [119]. At bath pH value 9, the Ni-P-Al_2_O_3_ coating developed enhances the metal surface hardness by 184% and decreases the rate of corrosion by 90%. At the same pH value, Ni-P-ZnO developed a 118 percent improved substrate hardness and diminished the rate of corrosion by 96% [120]. When the EN solution is heated to 85 °C and the pH is 8, the Ni-Fe-P coating has the best corrosion resistance [121].

### 4.2. Surfactant Effect on Ni-P Composite Coatings Properties

By adding a surfactant into the electroless solution, the properties of the coatings are considerably altered. With the presence of cationic surfactant, the coating formed has a minimum surface roughness. A mixture of the amorphous and crystalline nanostructures of the coating completely transformed to the crystalline phase in the presence of both surfactants, resulting in an increase in microhardness of approximately 50% [122,123,124,125]. Phosphorus presence improves the amorphous phase of the coating, resulting in improved corrosion resistance. An anionic surfactant is responsible for the maximum and homogeneous deposition of Al_2_O_3_, CNT, and TiC particles found in Ni-P composite coatings. Uniformly reinforced NPs in the Ni lattice in the existence of an anionic surfactant decrease the pores present in the coatings, resulting in improved corrosion resistance [126,127,128].

In the cationic surfactant presence, the electroless bath is more stable than nonionic and anionic surfactants. As a result, as the cationic surfactant concentration in the EN solution increases, the deposition rate of PTFE and zirconia NPs increases, resulting in a surface topography change and a smoother surface in the coating. Particle distribution is more homogeneous in the Ni matrix with the existence of cationic surfactant, which acts as an active barrier against the diffusion of corrosive ions, which results in superior corrosion protection to the Ni-P-PTFE and Ni-P-nanoZrO_2_ coatings formed from the cationic surfactant bath [129]. The impedance to corrosion of Ni-P-Al_2_O_3_ coatings increases up to 20 mg/L cationic surfactant (CTAB) concentration. Beyond this amount, a decline in the trend is observed as a result of the increased porosity of the coatings due to the nonhomogeneous distribution of incorporated particles [60]. The maximum microhardness value and corrosion protection capability observed in Ni-P-TiO_2_ coating is created in the presence of cationic surfactant [130]. Compared with the cationic surfactant, the hardness and wear resistance of the nano diamond-reinforced Ni-P coating are at their maximum in the presence of SDS surfactant [131]. The Ni-P-C_3_N_4_ coating generated from the nonionic surfactant has the highest microhardness value of 600 HV. The hardness of coatings made from anionic and cationic surfactants is 10% and 18% lower than that of coatings made from polymeric surfactants [132].

## 5. Polyalloy Coatings

Polyalloy coatings are often created by incorporating various reinforcing elements into the Ni matrix to extend the mechanical and chemical properties. Electroless coatings’ characteristics are typically calculated by the amount of phosphorus or boron, as well as the inclusion of metal components such as Cu, Fe, Co, Mo, W, Zn, Sn, and others [133,134,135,136,137].

### 5.1. Microhardness and Wear

For as-plated conditions, Balaraju et al. noticed that the microhardness of the Ni-W-P-Al_2_O_3_ (6 g/L) and Ni-P-W coatings is a bit equal [138]. At all annealing temperatures, the hardness and thermal stability of Ni-W-P-Al_2_O_3_ coatings are larger than the Ni-W-P coatings. The precipitation of nickel crystallites and the coarsening of Ni_3_P grains at higher annealing temperatures (600 °C) diminishes the hardness of the deposits. A poly alloy Ni-Zn-P-TiO_2_ coating’s hardness is superior to the Ni-Zn-P ternary coatings. When tested on low-carbon steels, annealed at 400 °C enhances the hardness of both coatings due to the production of meta-stable Ni_12_P_5_ and Ni_3_P phases [134]. To improve the microhardness, Ni-W-P was deposited on the mild steel. Before and after annealing at 400 °C, the hardness of Ni-W-P coating was better than Ni-Cu-P and Ni-W-Cu-P coatings. The grain size changes from 2.5 to 8.8 nm after copper reinforcement into the Ni-W-P deposit, resulting in a decrease in hardness. The addition of copper reduces the hardness of the coating by 7% [139]. The precipitation of Ni_3_P and the creation of a Ni-W solid solution at 400 °C thermal process temperature increases the hardness value of Ni-W-Cu-P and Ni-W-P coating by 64% and 65%, respectively. The reinforcement of MoS_2_ and WS_2_ particles into the Ni-P-SiC coating reduces its microhardness. However, higher microhardness is obtained with WS_2_ particles when compared to both particles. The wear failure is minimal in a Ni-P-SiC-WS_2_ coating due to the better lubricating properties of WS_2_ compared to MoS_2_ [140]. Liew et al. inspected the tribological properties of Ni-P/PTFE/Al_2_O_3_/MoS_2_ coatings in the presence of Mach 5 SL SAE 10 W-30 engine oil [141]. Admirable friction properties and low protection to wear were observed in the PTFE coating due to the low mechanical strength of the PTFE. Higher wear resistance and frictional values observed in the Al_2_O_3_particle-reinforced coatings are followed by MoS_2_ and PTFE particles co-deposited coatings.

Resistance to wear of the Gr-added Ni-P-SiC coatings rises with increasing Gr content. The highest wear loss of 25.69 × 10^−6^ g/m is noticed in the coating formed at 12 g/L Gr content [133]. The hardness of Ni-P-SiC-PTFE coating was 32% better than the Ni-P-PTFE coatings and 18% lower compared to the Ni-P coatings with SiC addition. The development of crystalline Ni_3_P at 400 °C heat treatment improves the hardness of three coatings. After annealing, the maximum hardness observed in the Ni-P-SiC coatings was 2.5 times that of the as-coated condition. Among the three coatings, a minimum friction coefficient (0.48) is noticed in the Ni-P-PTFE coatings [142]. The reinforcement of graphite particles into Ni-P-SiC coatings reduces its microhardness and wear damage before and after the annealing process [133]. The microhardness of a Ni-P-Cu coating increases from 500 HV to 1200 HV after introducing CNTs into the coating lattice [136]. The hardness and wear resistance of Ni-P coatings can be greatly increased by TiN or TiN+Re reinforcement after plating and thermal processing. The combination of Re and TiN significantly improves the coating performance [101].

### 5.2. Corrosion

The corrosion resistance of a Ni-P-W-Nb_2_O_5_ coating developed from an electroless solution containing 15 g/L Nb_2_O_5_ particles is maximum due to the high thermodynamic stability of the composite coating at that concentration [143]. The co deposition of copper into Ni-P and Ni-W-P does not much influence the P wt%, but the co deposition of W into Ni-P reduces the phosphorus concentration. At the high phosphorus content, the corrosion-defense ability of the Ni-Cu-P coating is at maximum [135]. In Ni-Zn-P-TiO_2_ coatings, TiO_2_ particles fill the insignificant pores in the deposited matrix, create a denser deposit, and reduce metal contact with corrosive media. This could result in a noticeable enrichment in the resistance to corrosion [134]. Compared to the Ni-P-TiO_2_ and Ni-P-Cg coatings, the pores in the Ni-P-Cg-TiO_2_ coating are smaller, which reduces the metal contact with the salty environment and results in higher corrosion resistance [144]. In Ni-P-Cu-CNT coatings, the presence of CNT makes the deposit more compact and provides better protection against corrosion [136]. The corrosion protection ability of Ni-P-Cu-PTFE in 1M HCl and 20% NaCl solution was improved over Ni-P-PTFE coatings [145]. The literature on electroless Ni-P composite coating for diverse substrates is listed in Table 1.

## 6. Ni-B, Ni-P-Ni-B Composite Coatings

### 6.1. Ni-B Coatings

Ni-B coatings are another type of nickel alloy coating in which borohydride is used as a reducing agent. Ni-B is the most popular coating after Ni-P due to its ability to improve mechanical and chemical properties. Ni-B and its alloy coatings are used on various materials to increase hardness, wear, and corrosion resistance are shown in Table 2.

#### 6.1.1. Deposition Rate and Microstructure of the Ni-B Coatings

Reducing the agent concentration, bath temperatures, and annealing temperatures influences the deposition rate, properties, and structure of the coating. Compared to high bath temperatures (95 ± 1 °C), coatings developed from the low bath temperatures (45 ± 1 °C) have minimum deposition rate. The coating rate of deposition increases by increasing the reducing agent and the highest deposition rate of 18–20 μm/h and 25–30 μm/h is observed at 0.8 g/L and 1.05 g/L NaBH_4_ concentrations from a high-temperature bath. At 1 g/L NaBH_4_ and 45 ± 1 °C bath temperature, the deposition rate of the coating is 10 μm/h [146]. The nature of the stabilizer employed in the deposition process also influences the deposition rate. The Ni-B coating rate of deposition (26 μm/h) in the presence of a tungsten-based stabilizer is at maximum compared to nitride, sulphate (24 μm/h), and chloride stabilizer (20 μm/h) baths. The surface roughness of the coating in the existence of nitride stabilizer has a lower surface roughness than the other stabilizers (0.34 μm). In the presence of four stabilizers, the morphology of the Ni-B coating seems like a cauliflower structure [147]. Due to the increased boron deposition rate at higher reducing-agent concentrations, the amorphous phase of the Ni-B coating increased [148,149]. The amorphous nature of the deposit is changed to crystalline Ni and Ni_3_B after the annealing.

#### 6.1.2. Microhardness and Resistance to Wear of the Ni-B Coatings

The hardness of the Ni-B-coated AZ91D alloy is 10% more than the substrate due to higher adhesion strength. The wear rate in the coating on both materials is the same for the applied load [150]. The microhardness and wear loss in the Ni-B coating on mild steel did not change much at lower annealing temperatures. The microhardness of the deposited coating increases as the thermal process temperature rises. The development of crystalline Ni, Ni_2_B, Ni_3_B, and Ni_4_B_3_ phases at 350 °C and 450 °C annealing temperatures improves the microhardness of the coatings. Above this temperature, coarse grain formation makes the coatings softer. The increment in hardness following the annealing process results in good wear resistance [151,152]. Correa et al. found that the annealing time affects the microhardness of the deposited thin film [153]. A higher microhardness value of 15 Gpa is identified in the coating for a 5 h heat-treatment duration at 300 °C temperature. A further increase in time up to 48 h decreases the microhardness of the deposit due to the development of higher FCC nickel. Growth in FCC nickel content makes the coating softer, which results in lower microhardness. Algul et al. developed the electroless NiP, NiB, and NiBP coatings for hardness testing before and after heat treatment with two separate reduction agents. Before and after the annealing process, the coating hardness follows the following order NiP < NiB < NiBP [154].

### 6.2. Ni-B Composite Coatings

Composite Ni-B coatings are created by emphasizing the oxides, carbides, and nitride particles in the nickel matrix. Reinforcement of TiO_2_ nanoparticles to the Ni-B coating lattice enhances its hardness. The maximum microhardness, minimum wear loss, and friction coefficient are obtained by the addition of 2 g/L TiO_2_ NPs. Further addition causes the agglomeration of particles, and this agglomeration increases the distance among the particles. Thus, reducing the dispersion strength causes a reduction in the deposit’s mechanical properties [155]. The Ni-B-Al_2_O_3_ coating had a lower wear rate at all tested temperatures (25°C, 200 °C, 400 °C, and 600 °C [156]. The oxidation performance of the Ni-P, Ni-B, and Ni-B-W coatings at different annealing temperatures ranges between 600–800 °C and is characterized by Eraslan et al. [157]. In Ni-B coatings, an oxide film of a thickness of 6μm is less than the Ni-P coating oxide film thickness (8 μm), confirming that the resistance to oxidation of the Ni-B deposit is superior to the Ni-P annealed at 700 °C. The presence of W in a Ni-B-W coating reduces the diffusion of iron into the coating surface and defers the creation of iron oxide at 700 °C annealing temperature. From the above, we can conclude that Ni-B-W has a higher oxidation resistance among the three coatings. Co deposited SiC and Si_3_N_4_ retain the anodic dissolution reaction by minimizing their effective metal interaction with the corrosive medium, which results in enrichment in the corrosion protection of the Ni-B composite coatings [158,159]. A Ni-B-CeO_2_ coating created at optimal CeO_2_ particle concentration (10 g/L) offers the highest wear resistance and microhardness than the other concentrations [160].

### 6.3. Ni-P-Ni-B Duplex Coatings

Compared to Ni-P coatings, Ni-B coatings have higher hardness and less wear damage and corrosion protection. Researchers have tried Ni-P-Ni-B duplex coatings by using a dual bath to achieve ideal properties [161,162,163,164]. The resistance to wear and hardness of multilayer Ni-B-Ni-P coatings is lower than Ni-B coating but better than Ni-P coating. The concentrations of phosphorus and boron determine the hardness of Ni-P and Ni-B coatings. The hardness of the Ni-P deposit decreases as the phosphorus level increases, while the hardness of the Ni-B coating increases as the boron percentage increases. In Ni-B coatings, boron forms a solid solution in the Ni domain, increasing the hardness of the deposit. After annealing, Ni-B coatings have higher hardness than Ni-P coating. The lower hardness value of Ni-P after heat treatment than Ni-B may be due to increased grain size and the Ni_3_P precipitate being smoother than the Ni_3_B precipitate. Ni-B coatings have high microhardness due to the formation of rich coherent boron precipitates in the nickel matrix. Therefore, Ni-B outer layer duplex coatings provide better hardness and wear resistance than Ni-P outer layer duplex coating [152,154]. When compared to single-layer coatings, Ni-P outer layer duplex coating offers superior corrosion protection. After annealing, the formation of crystalline Ni_3_P and Ni_3_B in both layers enhances the hardness and resistance to corrosion further [165,166,167]. Crystalline Ni, Ni_3_P, and Ni_3_B precipitates start to form after thermal process in the duplex coatings. The average grain size of the Ni-P coating before and after the annealing process is 1nm and 48 nm [168]. Vinod et al. found that the average grain size of a Ni-P deposit before and after the heat-treatment process was 42 nm and 24 nm [125]. Hasan et al. observed that the average grain size of a Ni-P and Ni-B coating was 15 nm and 10 nm [154]. After the heat-treatment process, improving the grain size improves the microhardness of the deposit [68]. The average grain size of Ni_3_B in a Ni-B coating ranges from 20 nm to 100 nm, which increases the hardness of the deposit [153]. The grain size of the Ni-B coating increases from 10 nm to 17 nm after the annealing process [159]. A duplex Ni-Zn-Cu-P/Ni-P coating improves the microhardness of magnesium alloys by up to 400%. The presence of Zn in the alloy lattice improves the grain structure, resulting in a 4% improvement in microhardness. A decrease in the potential gap between the coatings and the substrate mixture results in a positive shift in the corrosion potential, resulting in a higher resistance to corrosion for the dual-layer coating [162]. The co-deposition of B_4_C into the Ni-B lattice increases the corrosion protection capability of Ni-B coatings [164]. The corrosion resistance of the Ni-P-Ni-B-B_4_C duplex coating is better than that of the Ni-P coating due to the superior electrochemical resistivity of the B_4_C particles. The Ni-P/Ni-Mo-P duplex coating has a lower porosity (1.2 spot/cm^2^) than Ni-P and Ni-Mo-P single-layer coating, resulting in superior corrosion resistance. After the effective deposition of the Ni-P/Ni-Mo-P duplex coating, the corrosion-protection performance of the substrate surface increased to 98.5% [169]. The presence of harder SiC and electrochemical resistive ZnO nanoparticles in the Ni-P-ZnO/Ni-P-SiC duplex coating greatly improves the mild steel substrate’s hardness and corrosion resistance [170]. Jiaan Liu et al. used a combined microarc oxidation (MAO) and EN technology to develop a duplex coating on a friction stir welded (FSW) AZ31 alloy joint. The mechanical interlocking of the EN top layer with the MAO layer increases the MAO–EN interface bonding. The MAO reduced the possibility of galvanic corrosion between the Ni deposit and the Mg surface, whereas the EN top layer served as a seal for the MAO layer [171]. Mild steel substrate surface hardness and tribological performance were improved with duplex electroless Ni-P/Ni-Cu-P coatings before and after a 400 ℃ heating procedure. Since copper promotes the creation of a passive layer, the coating with Ni-Cu-P as the outer layer has greater corrosion resistance [172]. Resistance to corrosion of the NiP/TiN coatings formed at various pH levels (2, 7, 12) demonstrated a 25 times lower corrosion-current density in an alkaline environment compared to an acidic solution, confirming that the corrosion resistance of the Ni-P/TiN multi pass coatings is greater than that of aluminium and single TiN coatings [173].

**Table 2:** The literature on Ni-B composite coatings on various substrate materials.

S. No.	Coating	Mild Steel	Steel	Mg Alloy	Carbon Steel	Ref. No.
1	Ni-B	A	–	–	–	[147]
2	Ni-B	A	–	–	–	[152]
3	Ni-B-TiO_2_	A	–	–	–	[155]
4	Ni-B-W	A	–	–	–	[157]
5	Ni-B-SiC	–	–	–	C	[158]
6	Ni-B-Si_3_N_4_	E	–	–	–	[159]
7	Ni-B-CeO_2_	–	–	–	A	[160]
8	Ni-P-Zn-Cu-P/Ni-P	–	–	C	–	[162]
9	Ni-P/Ni-B-B_4_C	–	C	–	–	[164]
10	Ni-P-Ni-B	–	–	E	–	[165]
11	Ni-B-Ni-P	–	–	E	–	[166]

Note: A—Improvement in hardness and wear resistance, B—Improvement in resistance to corrosion, C—Improvement in hardness and corrosion resistance, D—Improvement in wear and corrosion resistance, and E—Improvement in wear and reduction in corrosion resistance.

## 7. Cu-P Composite Coatings

Copper’s unusual properties, including high ductility, malleability, thermal and electrical conductivities, and ease of machining, led to its selection as a matrix material. For the deposition of Cu films, the electroless copper (EC) method is a popular electrochemical technique. Due to its high thermal conductivity, it is widely used in the power sector, machinery production, and aerospace plane airframes. These coatings recommend a wide range of uses for conductive fabrics, relay blades, contact supports, and lead wires, and due to their high electrical conductivity and mechanical strength, electrode materials are ideal for spot welding. Copper formulation and plating techniques are being developed in response to the high demand for these applications [174,175]. In an electroless copper bath, Cu-P and composite coatings are created. Copper sulfate (as a copper source), sodium hypophosphite (as a reducing agent), sodium citrate (as a complexing agent), and boric acid are all added to the bath (as a buffering agent). The electroless copper solution was run at 88–92 °C and maintained at a pH range of 9–11. To achieve the required Cu-P coating, the materials to be coated were immersed in the solution for 3 h. Various secondary particles are added to the electroless solution at macro-, micro-, and nanoscales to generate Cu-P composite coatings. During the deposition process, magnetic stirring was utilized to keep particles suspended [176]. A few studies also attempted to pick up the thermal characteristics of the metal surface using Cu-based coatings [175,177]. The microhardness of the Cu-P coating can be increased to 47% by introducing hard SiC particles into the Cu lattice. The lamellar structured Cg deforms easily when loaded, resulting in a 33% decrease in microhardness for the Cu-P-Cg coating [178]. Moderate microhardness is observed in Cu-P-Cg-SiC coatings. The increasing order of microhardness is as follows: Cu–P–SiC > Cu–P > Cu–P–Cg–SiC > Cu–P–Cg > carbon steel. High hardness results in good wear resistance. Therefore, wear loss in a Cu-P-SiC coating is minimal compared to other coatings due to high hardness. The reinforcement of hard SiC particles into the Cu-P matrix increases the coefficient of friction, while the addition of lubricating graphite particles to the Cu-P matrix decreases the coating coefficient of friction. The introduction of SiC particles into the Cu-P and Cu-P-Cg lattice improves the corrosion resistance of the coating. The size of the SiC particles affects the properties of the Cu-P composite coating. The distribution of SiC particles at the nano level is more homogeneous in the Cu lattice than at the microlevel, resulting in a 33% improvement in the hardness of the Cu-P-nano SiC coating. Decreasing the SiC particle size from micron to nano increases the Cu-P composite coating-corrosion potential by 11%, confirming that Cu-P-nano SiC coatings have superior corrosion protection capability. Nano SiC particles effectively fill the micropores in the Cu-P matrix, inhibiting the diffusion of chloride ions along the interface and resulting in high corrosion resistance [179]. SiC particle reinforcement into the Cu-P lattice increases the concentration of SiC particles in the plating solution up to 5 g/L. Above this value, less deposition is observed due to the agglomeration of particles in the solution. Co deposited SiC NPs oppose the plastic deformation of the Cu alloy, resulting in the highest hardness and minimum wear and corrosion for the Cu-P coating [175,177,178,179,180]. The corrosion rate of mild steel in a 1 mol HCl solution can be significantly reduced by using Cu-P coating [181].

## 8. Applications and Recent Development of Electroless Coatings

### 8.1. Applications of Electroless Coatings

**Figure 4:**
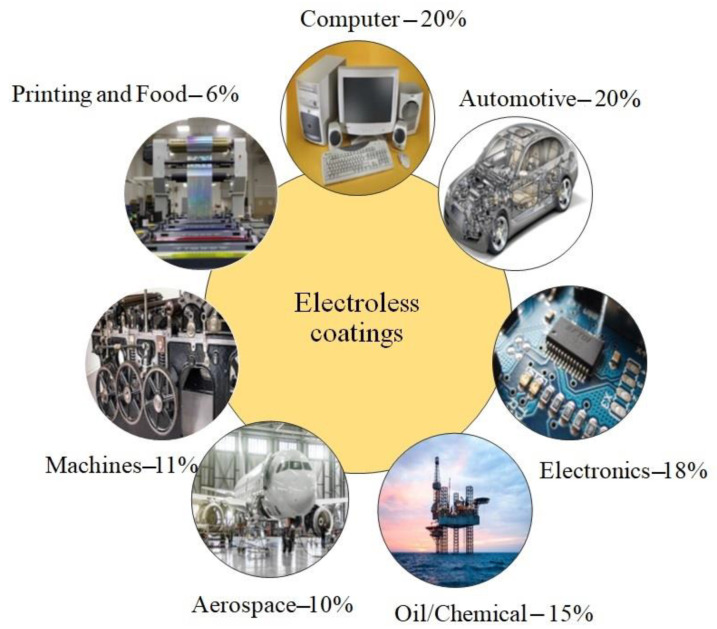
Percentage of utilization of the electroless coating in various fields [27,52,56,85].

The electroless method differs from other surface-coating methods due to its unique properties, such as uniform thickness. Electroless nickel coatings are ideal for steel, aluminium, copper, and polymers because they improve hardness and wear and corrosion resistance. The percentage of utilization of electroless coatings in various sectors is indicated in Figure 4.

Various parts such as steel mould used in the glass industry, differential pinion shafts, pistons, cranks, rotating shafts, bearing surfaces, cylinder bores, and viscous coupling plates in the automobile industry undergo high wear. Similarly, feeds and guides, fabric knives, spinnerets, loom ratchets, and knitting needles in the textile industry experience high wear due to the rapid movement of fibers. So, the components that are used in the automobile and glass industries require a coating with the lowest wear rate. The harder coating provides good wear resistance [50,57]. Electroless nickel is applied to the aluminium components used in aircraft or spacecraft to improve wear resistance and corrosion resistance. Electroless nickel is also used to cover printed circuit boards, light alloy dies, nuclear reactor components, and radar waveguides. Since a Ni-P/B composite coating is more durable, it can also be employed in the automotive, chemical, and textile industries. The carburetor and fuel injection systems used in the automobile industry require corrosion resistance during alternative fuel usage. Likewise, the hydraulic actuator splines, landing-gear components, engine mounts, engine-oil feed tubes, etc. used in the aerospace industry also require corrosion protection. From the above studies, it is confirmed that electroless nickel coatings offer excellent resistance to corrosion [27,89]. The industrial applications of electroless coatings are represented in Table 3.

### 8.2. Recent Development in the Electroless Coatings

The bath composition, pH value, and operation temperature all influence the electroless nickel coating’s properties. The deposition rate is a critical characteristic that has a considerable impact on the electroless deposition’s properties. Fora few decades, various techniques, such as polarization, gravimetry, and electrochemical quartz crystal microbalance, have been employed to evaluate deposition rate. The continuous noise resistance calculation (CNRC) method is a relatively new method for determining the deposition rate. Since the deposition rate determined using the CNRC technique closely matches the gravimetric data, CNRC is considered a useful tool for online electroless deposition-rate evaluation [183]. Electroless Ni-P deposits have been created in the presence of a magnetic field in recent years. During the autocatalytic reaction, a magnetic field is precisely supplied to the bottom of the sample to adsorb the metallic nickel particles produced. This is expected to reduce nickel waste and alter crystallization behaviour. Using a magnetic field in the deposition process increased the deposition efficiency by 20% [184].

A frequently used method to improve the properties of electroless nickel plating is the traditional heat-treatment process. In recent years, the nitriding postdeposition treatment technique to improve the properties of electroless nickel coatings has attracted much attention. Plasma nitride high boron Ni-B coatings have higher microhardness than annealed Ni-B coatings. Wear resistance is 14 times higher in plasma nitride Ni-B coatings compared to annealed [182]. To improve the qualities of heat-treated electroless nickel coatings, the air-quenching method is used to cool them. Water quenching has been utilized to cool heat-treated nickel coatings in recent years. When compared to air quenching, the water-quenching technique considerably improves the coating’s characteristics. The deposit’s superior properties are due to its finer crystallite size and the production of intermediate phases in the crystal structure during water quenching [185]. Improving adherence at the coating–substrate interface can be as simple as fabricating textured substrate surfaces. The radial ultrasonic vibration-aided turning (RUVT) technique is used to produce complex micro textures on substrate surfaces before they are coated. The test findings show that the created micro textures increased coating adherence. As a result, RUVT’s intricate micro textures make it easier to attach a coating to a stainless-steel substrate [186].

Electroless Ni coatings have been tested on the natural fibre used in an epoxy polymer matrix to improve composite characteristics in recent years. Coated fibre–composite materials showed an increase in tensile, flexural, and impact strength [187,188]. To reduce oxidation and mass loss, an electroless copper coating is deposited on the carbon fibers used in the manufacture of magnesium matrix composites. The oxidation and mass loss of carbon fibers used in the manufacture of Mg composite materials are dramatically reduced by electroless copper coating [189]. By increasing the Ni^2+^ percentage in the deposit solution, the bonding capacity and copper deposition on carbon fibre can be improved [190]. Due to their high strength-to-weight ratio and lightness, magnesium alloys are widely used in electronic items, automobiles, and aircraft. The poor corrosion and wear resistance of Mg alloys is a major barrier to their use in many industries. Ni-P-SiC coatings provide improved corrosion protection for Mg alloys in a 3.5% NaCl solution, making the use of Mg and similar alloys more viable in hostile environments [189].

## 9. Conclusions and Future Developments

Researchers are examining how different soft and hard ceramic micro- or nanoparticles affect the characteristics of the composite coating Ni-P/B. The effect of process parameters on the characteristics, behaviour, and attributes of EN coating, including the agent and nickel-source concentration, solution temperature, pH range, and type of surfactant added to the bath. The following findings are reached based on the body of extant literature. The composition of the co-deposited particle and surfactant, the stabilizer concentration, and the particle size reduction all have an impact on the mechanical behaviour of EN composite coatings. The features of EN coating, such as microhardness, wear resistance, and corrosion protection, are considerably enhanced by the phase transition of the Ni matrix. A phase transition happened when the coatings were heated to the ideal temperature of 350 to 450 °C. The characteristics of the coating are decreased by annealing at temperatures above 450 °C because the matrix is amorphous. When the concentration of the reducing agent is increased to optimal levels, the properties of the composite coating are considerably altered; nevertheless, excessive concentrations are not advised due to bath disintegration. The type of particles reinforced in the coating matrix has a significant impact on the deposit’s microhardness, wear resistance, and corrosion resistance. Hard ceramic particles, Al_2_O_3_, SiC, TiO_2_, SiO_2_, CNT, and TiC, are reinforced into a Ni matrix to increase EN coating hardness while also providing wear resistance and corrosion protection. When lubrication is essential, soft particles like graphite and PTFE are utilized. Coating characteristics are influenced by particle size. EN composite coating is created by microlevel integration of particles into an alloy matrix to achieve superior hardness, wear, and corrosion resistance. Co-deposited coating with nanoscale particles has an excellent surface quality and a low coefficient of friction. A ceramic particle and surfactant combination is highly advised to provide improved hardness and corrosion protection capacity. Multiple pass coatings with different combinations have been demonstrated and recommended to get improved characteristics. Electroless nickel and copper coatings for ceramic and glass materials have new uses. Emerging coating technologies, like electroless nickel and copper coatings, are needed to combat the world’s feared throwaway culture and restore the usability of those parts by improving their anticorrosion and wear characteristics. To make electroless nickel–boron plating more ecologically friendly, there is still much study to be done. Hard NP reinforcement enhances hardness and wear resistance, whereas soft NP reinforcement reduces hardness and coefficient of friction. Due to this, future research can use both smooth and rough particle types in Ni-B coatings simultaneously, increasing the tribological qualities.

## Figures and Tables

**Table 1:** The literature on electroless Ni-P/Ni-P composite coatings on various substrate materials.

S. No.	Coating	Al Alloy	Mild Steel	Steel	Mg Alloy	Carbon Steel	Copper	Ref. No.
1	Ni-P	–	A	–	–	–	–	[25]
2	Ni-P	D	–	–	–	–	–	[31]
3	Ni-P	–	–	D	–	–	–	[33]
4	Ni-P	–	–	A, B	–	–	–	[35]
5	Ni-P-SiC	–	C	–	–	–	–	[51]
6	Ni-P-SiC	D	–	–	–	–	–	[70]
7	Ni-P-W	–	E	–	–	–	–	[72]
8	Ni-P-Al_2_O_3_	–	A	–	–	–	–	[27]
9	Ni-P-CNT	–	–	–	–	–	C	[80]
10	Ni-P-Si_3_N_4_	–	–	–	–	A	–	[84]
11	Ni-P-TiN	–	–	–	–	E	–	[86]
12	Ni-P-ZnO	–	C	–	–	–	–	[87]
13	Ni-P-TiO_2_	C	–	–	–	–	–	[89]
14	Ni-P-SiC	–	–	–	D	–	–	[96]
15	Ni-P-W	–	–	A	–	–	–	[98]
16	Ni-P-SiC	–	B	–	–	–	–	[107]
17	Ni-P-CNT	–	B	–	–	–	–	[108]
18	Ni-P-TiO_2_	–	–	–	B	–	–	[111]
19	Ni-P-ZnO	–	D	–	–	–	–	[120]
20	Ni-P-Zn-TiO_2_	–	C	–	–	–	–	[134]
21	Ni-P-SiC-WS_2_	–	–	C	–	–	–	[140]
22	Ni-P-Cu-W	–	A	–	–	–	–	[143]
23	Ni-P-C_g_-TiO_2_	–	–	–	–	E	–	[144]

Note: A—Improvement in hardness and wear resistance, B—Improvement in corrosion resistance, C—Improvement in hardness and corrosion resistance, D—Improvement in wear resistance and corrosion resistance, and E—Improvement in wear resistance and reduction in corrosion resistance.

**Table 3:** Industrial applications of electroless coatings [27,107,161,182].

Type of Work	Type of Work with Detailed Reason for Use
Food industry	Protect the packing equipment such as bearings, rollers, conveyer systems, hydraulics, and gears in meat processing from corrosion
Valves and flow-control devices	Resists chemical corrosion as well as provides hardness and wear resistance.
Mud-pump bodies	Protects against corrosion by drilling mud
Foundry tooling	Improves release characteristics and resists chemical corrosion
Chemical-process industry	Protects filters, heat exchanger pumps, tanks, and pipe fittings from corrosion by brine
Printed circuits in electronic industry	Provides ease of solderability
Turbine parts	Produces coating uniformity, which preserves the balance of the turbine
Gears and gear assemblies	Provides hardness and wear resistance as wellas high corrosion
Bearing journals, servo valves, compressor blades, engine mounts, landing gear, and hydraulic and manifold systems in the aerospace industry	Makes possible accurate control of dimensions, uniform coverage inall areas, and good hardness, wear, and corrosion resistance.

## Data Availability

Not applicable.
